# Inherited unbalanced reciprocal translocation with 3q duplication and 5p deletion in a foetus revealed by cell-free foetal DNA (cffDNA) testing: a case report

**DOI:** 10.1186/s40001-021-00535-5

**Published:** 2021-06-29

**Authors:** Taccyanna M. Ali, Emilia Mateu-Brull, Nuria Balaguer, Camila Dantas, Haline Risso Borges, Mariana Quintans Guerra de Oliveira, Lorena Rodrigo, Inmaculada Campos-Galindo, Roser Navarro, Miguel Milán

**Affiliations:** 1 Laboratório Igenomix, Laboratório de Genética E Medicina Reprodutiva, Sao Paulo, Sao Paulo Brazil; 2 IGENOMIX Lab S.L.U., Parque tecnológico, Ronda Narciso Monturiol, 11B, Edificios Europark, 46980 Paterna, Valencia Spain; 3 Clínica HR—Santa Rita do Passa Quatro, Sao Paulo, Brazil; 4 Clínica Nanu—Santa Rita do Passa Quatro, Sao Paulo, Brazil

**Keywords:** Cell-free DNA, Copy number variations, Reciprocal translocation

## Abstract

**Background:**

Since 2011, screening maternal blood for cell-free foetal DNA (cffDNA) fragments has offered a robust clinical tool to classify pregnancy as low or high-risk for Down, Edwards, and Patau syndromes. With recent advances in molecular biology and improvements in data analysis algorithms, the screening’s scope of analysis continues to expand. Indeed, screening now encompassess additional conditions, including aneuploidies for sex chromosomes, microdeletions and microduplications, rare autosomal trisomies, and, more recently, segmental deletions and duplications called copy number variations (CNVs). Yet, the ability to detect CNVs creates a new challenge for cffDNA analysis in couples in which one member carries a structural rearrangement such as a translocation or inversion.

**Case presentation:**

We report a segmental duplication of the long arm of chromosome 3 and a segmental deletion of the short arm of chromosome 5 detected by cffDNA analysis in a 25-year-old pregnant woman. The blood sample was sequenced on a NextSeq 550 (Illumina) using the VeriSeq NIPT Solution v1 assay. G-band karyotyping in amniotic fluid only detected an abnormality in chromosome 5. Next-generation sequencing in amniocytes confirmed both abnormalities and identified breakpoints in 3q26.32q29 and 5p13.3p15. The foetus died at 21 weeks of gestation due to multiple abnormalities, and later G-band karyotyping in the parents revealed that the father was a carrier of a balanced reciprocal translocation [46,XY,t(3;5)(q26.2;p13)]. Maternal karyotype appeared normal.

**Conclusion:**

This case provides evidence that extended cffDNA can detect, in addition to aneuploidies for whole chromosomes, large segmental aneuploidies. In some cases, this may indicate the presence of chromosomal rearrangements in a parent. Such abnormalities are outside the scope of standard cffDNA analysis targeting chromosomes 13, 18, 21, X, and Y, potentially leading to undiagnosed congenital conditions.

## Background

Analysis of cell-free foetal DNA (cffDNA) in the maternal bloodstream is a non-invasive prenatal approach that enables clinical screening for aneuploidies, minimising risks and the need for invasive procedures [1–4]. Presence of cffDNA in the maternal bloodstream was initially described by Lo et al. [5] in 1997. cffDNA consists of 150–200-base pair (bp) fragments that originate mainly from the placenta and are released into the mother’s blood, detectable at about 4–5 weeks of gestation [6]. At about 10 weeks, the concentration of cffDNA reaches a sufficient level to provide adequate specificity and sensitivity to perform screening in prenatal care [7].

Since 2011, providers have used clinical cffDNA screening in maternal blood to detect the most frequently observed foetal chromosome aneuploidies, including Down syndrome (trisomy 21), Edwards syndrome (trisomy 18), Patau syndrome (trisomy 13), and sex chromosome aneuploidies like Turner syndrome (45,X) and Klinefelter syndrome (47,XXY) [8–10]. cffDNA analysis can also determine foetal rhesus D status [11] and detect some microdeletion syndromes such as 22q11 deletion syndrome, cri-du-chat syndrome (5p deletion), Wolf–Hirschhorn syndrome (4p deletion), Prader Willi or Angelman syndrome (15q deletion), Jacobsen syndrome (11q deletion), Langer–Giedion syndrome (8q deletion), and 1p36 deletion syndrome [12–14].

With new advances in next-generation sequencing (NGS) and bioinformatic analysis, the number of genetic conditions analysable by cffDNA screening is expanding. For instance, analysis of all 24 chromosomes [15–18] and even detection of some monogenic conditions [19] are currently available in the portfolio of some laboratories offering cffDNA analysis worldwide. One of the most interesting advances in analysis of cffDNA in maternal blood is detection of placental copy number variations (CNVs) of a relatively long size without increasing sequencing depth [20–25].

The ability to detect CNVs entails a new challenge for cffDNA analysis, enabling detection of new genetic conditions that have gone unnoticed in couples in which one member is a carrier of a structural rearrangement such as a translocation or inversion [26]. Here, we describe a case of two segmental aneuploidies initially detected by extended cffDNA analysis and confirmed in the foetus by invasive procedures. Karyotype of the parents revealed that the father carried a balanced reciprocal translocation.

## Case presentation

### Clinical report

A 25‐year‐old, G1P0, healthy pregnant woman was referred to the Clínica Nanu in Sao Paulo, Brazil, at a gestational age of 16 weeks. As routine practice, an ultrasound examination was performed to monitor the foetus’s developmental state. Ultrasonograms revealed a nuchal translucency (NT) of 7.12 mm and generalised oedema. The pregnant woman was 167 cm tall and weighed 59 kg and exhibited typical developmental milestones. She and her 29-year-old husband were not consanguineous. There was no family history of genetic disease, but her husband’s mother and half-sister had a history of miscarriage without a defined aetiology. Despite the abnormalities observed by ultrasound, the patient refused amniocentesis.

### Screening for aneuploidies by cffDNA analysis

**Fig. 1:**
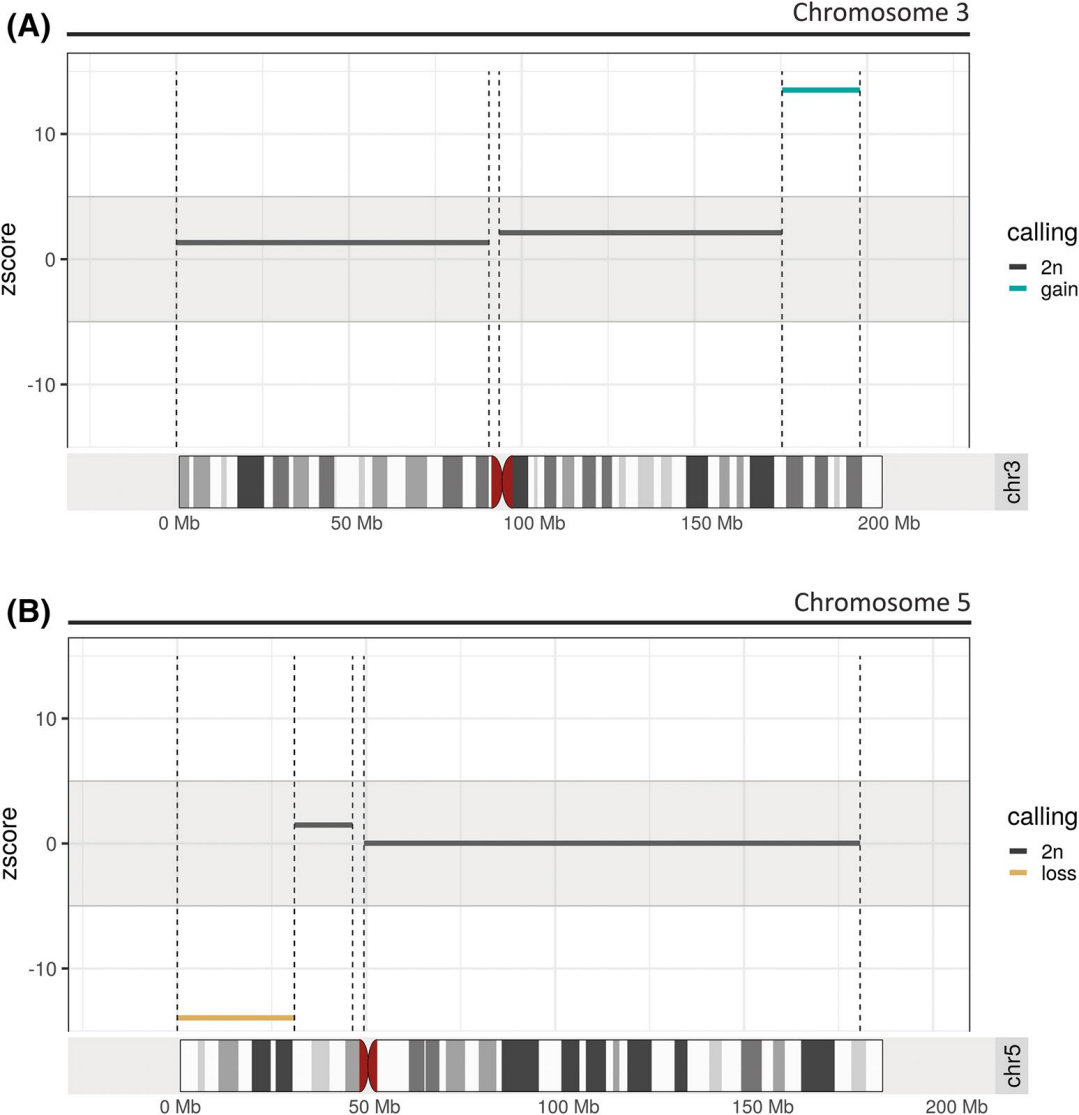
Results of partial aneuploidy screening performed with the VeriSeq NIPT Solution v1 assay and the WisecondorX software, using cffDNA from the maternal bloodstream. Z-score variations (vertical axis) for the different cytogenetic positions (horizontal axis) are shown. Note that the distances between the cytogenetic bands are presented in megabases (Mb). **A** Results showing a gain of approximately 25 Mb in the long arm of chromosome 3 (light blue bar) with a mean Z-score value of 13.5. **B** Results showing a loss of approximately 30 Mb in the short arm of chromosome 5 (orange bar) with a mean Z-score value of − 13.95

Extended cffDNA analysis in maternal blood was offered to screen for aneuploidies. The analysis was performed at Igenomix Brazil with the VeriSeq NIPT Solution v1 assay (Illumina). Briefly, cffDNA was obtained from 1‐mL plasma with a modified protocol of the QIAamp DNA Blood Mini Kit (QIAGEN GmbH, Hilden, Germany). Sample indexing and library preparation for sequencing were performed using TruSeqNano DNA Sample Prep Kits (Illumina Inc., San Diego, CA, USA). Libraries of all samples of a run were pooled, and paired‐end reads of 36 bp were sequenced in a NextSeq 500 system (Illumina Inc.). VeriSeqNIPT Assay Software v1 was used for the analysis of the aneuploidy status and foetal fraction. To detect partial abnormalities, WisecondorX (v1.1.5) was run following the default workflow [27], and visualisation was generated using ggplot2 [28] and ggbio [29] R packages. Results of the cfDNA analysis with WisecondorX showed two calls (|z-score|> 5) on chromosomes 3 and 5, compatible with a duplication of approximately 20 Mb (z-score = 13.5) in chromosome 3q (Fig. 1A) and a deletion of approximately 30 Mb in chromosome 5p (z-score = − 13.95) (Fig. 1B). To rule out the possibility of a sequencing artefact, cffDNA analysis was performed on a second blood draw, and the results were concordant. Results were discussed with the obstetrician, and the couple received genetic counselling.

### Chromosome analysis on amniotic fluid by karyotype

**Fig. 2:**
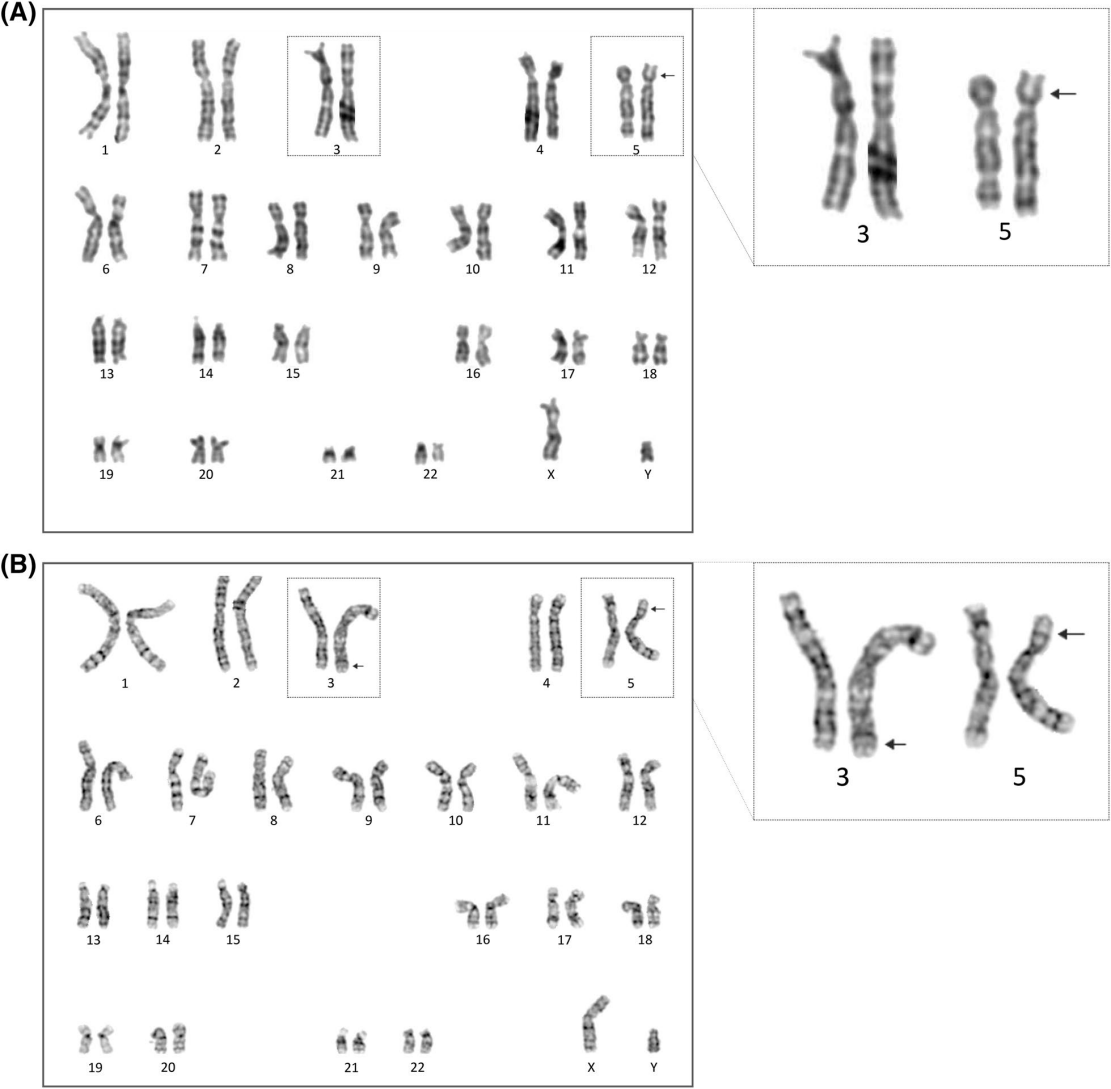
G-banded karyotype of cultured amniocytes (**A**) and paternal lymphocytes (**B**). Level of resolution of 400 and 550 bands, respectively

A subsequent ultrasound indicated spina bifida, severe cystic hygroma, and hydrops. Given the nature of both the ultrasound and cffDNA results, the patient agreed to have an amniocentesis to confirm the findings. Amniocentesis was performed of ultrasound at the Centro de Ultrassonografia, Medicina e Cirurgia Fetal FMFLA. Two samples of amniotic fluid were collected; one was sent to an external laboratory for karyotype analysis. Amniocytes were cultured in appropriate culture media, and 25 metaphases were analysed (G‐banding with a resolution of 400 bands). Cytogenetic analysis of amniocytes revealed an abnormal chromosome set corresponding to a cytogenetically male foetus showing an alteration in chromosome 5 but not in chromosome 3 (46,XY,der(5)) (Fig. 2A).

### Chromosome analysis on amniotic fluid by NGS

**Fig. 3:**
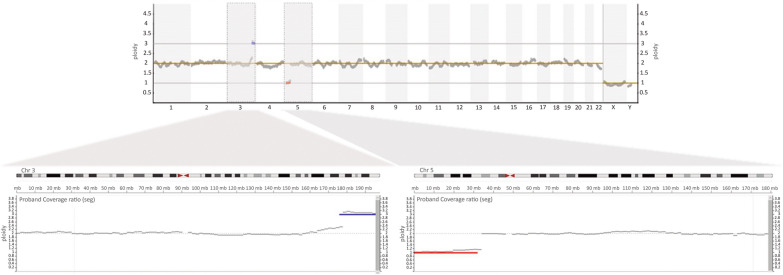
Detection of segmental unbalanced chromosomal aberrations by NGS from DNA extracted from amniocytes. Top graph shows results for all 24 chromosomes. Latter are represented in the X-axis, and copy number values are shown in the Y-axis. Typical copy number values regarding autosomal chromosomes are expected to be around 2. Single-copy gains of whole or partial segments of chromosomes display the copy number line around 3 (blue line). In comparison, single-copy losses of whole or partial segments of chromosomes have a copy number line around 1 (red line). In this case in particular, a terminal chromosome 3 gain (blue) and a terminal chromosome 5 loss (red) were observed. Lower graphs represent isolated chromosomes 3 and 5 and show a closer look at each chromosome's alterations. Chromosomal breakpoints were established at 3q26.32 and 5p13.3, involving a segmental gain of 22.1 Mb and a segmental loss of 32.0, respectively

A second aliquot of amniotic fluid was sent to Igenomix Brazil for rapid prenatal diagnosis by qfPCR, which detected no anomaly for chromosomes 13, 18, 21, or sex chromosomes. Given that the karyotype did not detect the alteration observed by cffDNA analysis in chromosome 3, we used genomic DNA from uncultured amniocytes to perform NGS to identify single-copy losses and gains of whole or partial segments of chromosomes. NGS was performed according to the protocol published by García-Pascual et al. [30]. With our procedure validated to detect aneuploidies > 10 Mb, we determined chromosomal breakpoints. Analysis revealed both alterations and chromosomal breakpoints at 3q26.32 and 5p13.3. These involved a segmental gain of 22.1 Mb of genetic material in chromosome 3 [(chr3:175865635_197962430) × 3; 3q26.32q29] and a segmental loss of 32.0 Mb in chromosome 5 [(chr5:10000_31993149) × 1; 5p15.33p13.3] (Fig. 3). According to data in UCSC, ClinVar, and Decipher databases, there are 230 genes in this chromosome 3 fragment, 44 of which are described in the OMIM database as related to a pathology. In the chromosome 5 fragment, 128 genes are described, 16 of which are related to a pathology.

The foetus died in utero at 21 weeks due to multiple malformations.

### Cytogenetic assessment of parents by karyotype

After confirmation of abnormalities in amniotic fluid, the couple received further genetic counselling. Karyotyping of peripheral blood (at a resolution of 550 bands) was performed to rule out a balanced rearrangement in either parent. The paternal karyotype showed a balanced translocation between chromosomes 3 and 5 [46, XY,t(3;5)(q26.2;p13)] (Fig. 2B), while the maternal karyotype revealed no apparent abnormality.

Taking into account all these findings, we established the result of the complete cytogenomic analysis for the foetus as:

46,XY,der(5)t(3;5)(q26.2;p13)dpat.seq[GRCh37] der(5)t(3;5)(q26.32;5p13.3)dpat.

NC_000005.10:g.pter_31993149delins[NC_000003.12:g.175865635_qter].

## Discussion and conclusion

Prenatal diagnosis of large subchromosomal CNVs in clinical practice still relies on invasive testing, such as karyotyping of placental or foetal genetic material through chorionic villus sampling (CVS) or amniocentesis [31]. Chromosomal microarray analysis (CMA) is a robust tool for detecting invisible small chromosomal deletions or duplications and is recommended as a first-tier diagnostic tool for some patients with well-defined syndromes [32]. During the prenatal period, CMA is strongly recommended for high-risk pregnancies, especially those with abnormal ultrasound with no alterations detected in the karyotype obtained from amniotic fluid or CVS [33]. In general, there is no recommendation for CMA in low-risk pregnancies with no ultrasound findings. Since abnormal CNVs can occur even in pregnancies without any structural abnormality observed in ultrasound [33], these genetic changes may constitute the first available evidence to detect a future newborn with congenital conditions or intellectual disabilities.

Since the initial clinical application of cffDNA analysis, the main objective has been to classify pregnant women with low- or high-risk for whole chromosome anomalies, especially chromosomes 13, 18, 21, X, and Y. However, improvement of the algorithms for data analysis used in the cffDNA screening now enables detection of other genetic changes. That includes CNVs, which in many cases are associated with maternal pathological processes such as gynaecological and haematologic tumours [34–37]. Correspondingly, increasing studies describe CNV alterations detected by cffDNA analysis during pregnancy and confirmed by invasive procedures. Wang et al. [37] described detection of subchromosomal abnormalities in chromosomes 13 and 21 derived from the mother with a balanced translocation [46,XX,inv(9)(p12q13),t(13;21)(q31.3;q21.3)]; Mei et al. [38] described a 10p15.3p13 duplication inherited from the father with a balanced translocation [46,XY,t(5;10)(q35.1;p13)]; Chen et al. [39] described a duplication in 18q11.32q21.2 and a deletion in Xp22.33p11 derived from a maternal reciprocal translocation; Zheng et al. [40] found a deletion in chromosome 21 of a foetus whose karyotype was 46,XN,del(21)(q11.2q22.1); and finally, Liu et al. [41] described the concomitant occurrence of 8q duplication and 13q deletion in a foetus derived from a maternal balanced translocation. Similar cases have been reported for patients undergoing cffDNA analysis in maternal blood. Recently, Yin et al. [42] described detection of CNVs by cffDNA testing with 32 of 48 suspicious cases confirmed with amniocentesis karyotyping.

Here, we describe a case in which cffDNA analysis classified the pregnancy as high-risk for foetal chromosomal abnormalities (a 3q segmental duplication and 5p segmental deletion). Initially, only the abnormality in chromosome 5 was confirmed by karyotyping of amniotic fluid. The recommended resolution in routine karyotyping when analysing amniocytes is 400 bands [43], with a theoretical resolution of 5–10 Mb. However, the actual resolution depends on different factors (e.g., type of bands affected, efficiency in chromosome preparation). In many cases, large CNVs could sometimes be missed [33, 44, 45]. This case highlights that, in the absence of a CMA, optimal resolution for the karyotype should have been 550 bands [46]. A subsequent analysis using NGS in DNA from uncultured amniocytes confirmed the cffDNA findings, setting the chromosomal breakpoints at 3q26.32 and 5p13.3. These imbalances were later traced to a balanced reciprocal translocation in the father (Fig. 2B), establishing the karyotype for the foetus as 46,XY,der(5)t(3;5)(q26.2;p13)pat.

The 3q duplication [dup(3q)] syndrome is rare, usually diagnosed after birth [47, 48], and is characterised by anomalies in the limbs and possible association with internal malformations, growth deficiency, dysmorphic face, and cognitive deficits [49]. Pasińska et al. [50] reported a prenatal diagnosis of 3q duplication syndrome in a foetus with enlarged skin oedema up to the sacral and pericranial regions with a NT of 8 mm. Interestingly, we observed oedema and a similar NT value in the ultrasound examination. The critical region responsible for the typical phenotype of dup(3q) syndrome was mapped to region 3q26.3q27.7, which contains many genes (e.g., *NLGN1, BCHE, TNIK, SOX2, Map6D1*) characterised by high expression during foetal brain development [51, 52]. The CNV we report here involves the 3q29 region associated with microcephaly, moderate cognitive deficits, and other abnormalities [53] that characterise 3q29 microduplication syndrome (MIM 611936). As reported by Abreu-González et al. [54], some patients with dup(3q) syndrome also have duplicated 3q29, so this region also could contribute to the phenotype.

Deletions involving the short arm of chromosome 5 are associated with cri-du-chat syndrome (MIM 123450), which is characterised by microcephaly, craniofacial anomalies, catlike crying during the first years of life, psychomotor delay, and possible presence of heart malformations or other anomalies [49]. Gu et al. [55] reported the same subchromosomal deletion (5p13.3p15.33) spanning ~ 26.22 Mb in two probands with no diagnosis of cri-du-chat syndrome during infancy but presenting developmental delay and dysmorphic and autistic features.

Expanding the scope of cffDNA screening analysis to CNV detection is interesting because many people carry an undetected balanced reciprocal translocation (0.2% of newborns). In other words, 1 in every 250 couples has a high probability of generating embryos with unbalanced rearrangements, some of which may result in children born with congenital abnormalities. Notably, cffDNA analysis is currently not accurate enough to detect all CNVs at any size and at any foetal fraction. However, detection of large subchromosomal aberrations is feasible [26]; indeed, this approach is described widely in the literature and may identify cases that otherwise go unnoticed. In the case of parents who know they are carriers of balanced translocations and ask for a non-invasive screening approach, some promising data have been published but more research is needed to establish the ability of non-invasive prenatal testing to detect imbalances [56]. According to Srebniak et al. [26], cffDNA screening could be the second-best choice when one parent carries a balanced chromosome aberration and refuses invasive testing because of difficulty achieving a viable pregnancy. Nonetheless, sufficient pre-test counselling and analysis of previous literature on the translocation to determine whether the imbalance is within the platform's detection limits are still required.

In conclusion, the case described here provides further evidence of the possibility of classifying pregnancies by cffDNA analysis as high-risk not only for common trisomies, but also for genetic deletions and duplications. In some cases, this classification may indicate risk of a parent carrying a balanced rearrangement, which would require genetic and reproductive counselling.

## Data Availability

The data that support the findings of this study are available from Igenomix Lab S. L.U., but restrictions apply to the availability of these data, which were used under license for the current study and thus are not publicly available. Data are available from the authors upon reasonable request and with permission of Igenomix, S.L.

## Abbreviations

bp: Base pair
cffDNA: Cell-free foetal DNA
CMA: Chromosomal microarray analysis
CNV: Copy number variation
CVS: Chorionic villus sampling
NGS: Next-generation sequencing
NT: Nuchal translucency

## References

[1] Bianchi DW, Parker RL, Wentworth J, Madankumar R, Saffer C, Das AF (2014). DNA sequencing versus standard prenatal aneuploidy screening. N Engl J Med.

[2] Warsof SL, Larion S, Abuhamad AZ (2015). Overview of the impact of noninvasive prenatal testing on diagnostic procedures. Prenat Diagn.

[3] Hui L, Hutchinson B, Poulton A, Halliday J (2017). Population-based impact of noninvasive prenatal screening on screening and diagnostic testing for fetal aneuploidy. Genet Med.

[4] Andari MVC, Bussamra SLC, Tedesco TGD, Peixoto PAB, Pares PDBS, Braga A (2020). Noninvasive prenatal testing: benefits and limitations of the available tests. Ceska Gynekol.

[5] Lo YM, Corbetta N, Chamberlain PF, Rai V, Sargent IL, Redman CW (1997). Presence of fetal DNA in maternal plasma and serum. Lancet.

[6] Thomas MR, Tutschek B, Frost A, Rodeck CH, Yazdani N, Craft I (1995). The time of appearance and disappearance of fetal DNA from the maternal circulation. Prenat Diagn.

[7] Rafi I, Hill M, Hayward J, Chitty LS (2017). Non-invasive prenatal testing: use of cell-free fetal DNA in Down syndrome screening. Br J Gen Pract.

[8] Nicolaides KH, Syngelaki A, Ashoor G, Birdir C, Touzet G (2012). Noninvasive prenatal testing for fetal trisomies in a routinely screened first-trimester population. Am J Obstet Gynecol.

[9] Zimmermann B, Hill M, Gemelos G, Demko Z, Banjevic M, Baner J (2012). Noninvasive prenatal aneuploidy testing of chromosomes 13, 18, 21, X, and Y, using targeted sequencing of polymorphic loci. Prenat Diagn.

[10] Palomaki GE, Deciu C, Kloza EM, Lambert-Messerlian GM, Haddow JE, Neveux LM (2012). DNA sequencing of maternal plasma reliably identifies trisomy 18 and trisomy 13 as well as Down syndrome: an international collaborative study. Genet Med.

[11] Aykut A, Onay H, Sagol S, Gunduz C, Ozkinay F, Cogulu O (2013). Determination of fetal rhesus d status by maternal plasma DNA analysis. Balkan J Med Genet.

[12] Grace MR, Hardisty E, Dotters-Katz SK, Vora NL, Kuller JA (2016). Cell-free DNA screening: complexities and challenges of clinical implementation. Obstet Gynecol Surv.

[13] Suciu ID, Toader OD, Galeva S, Pop L (2019). Non-invasive prenatal testing beyond trisomies. J Med Life.

[14] Martin K, Iyengar S, Kalyan A, Lan C, Simon AL, Stosic M (2018). Clinical experience with a single-nucleotide polymorphism-based non-invasive prenatal test for five clinically significant microdeletions. Clin Genet.

[15] Scott F, Bonifacio M, Sandow R, Ellis K, Smet ME, McLennan A (2018). Rare autosomal trisomies: important and not so rare. Prenat Diagn.

[16] Benn P, Malvestiti F, Grimi B, Maggi F, Simoni G, Grati FR (2019). Rare autosomal trisomies: comparison of detection through cell-free DNA analysis and direct chromosome preparation of chorionic villus samples. Ultrasound Obstet Gynecol.

[17] Chatron N, Till M, Abel C, Bardel C, Ramond F, Sanlaville D (2019). Detection of rare autosomal trisomies through non-invasive prenatal testing: benefits for pregnancy management. Ultrasound Obstet Gynecol.

[18] Pertile MD, Halks-Miller M, Flowers N, Barbacioru C, Kinnings SL, Vavrek D (2017). Rare autosomal trisomies, revealed by maternal plasma DNA sequencing, suggest increased risk of feto-placental disease. Sci Transl Med.

[19] Scotchman E, Chandler NJ, Mellis R, Chitty LS (2020). Noninvasive prenatal diagnosis of single-gene diseases: the next frontier. Clin Chem.

[20] Liu H, Gao Y, Hu Z, Lin L, Yin X, Wang J (2016). Performance evaluation of NIPT in detection of chromosomal copy number variants using low-coverage whole-genome sequencing of plasma DNA. PLoS ONE.

[21] Li R, Wan J, Zhang Y, Fu F, Ou Y, Jing X (2016). Detection of fetal copy number variants by non-invasive prenatal testing for common aneuploidies. Ultrasound Obstet Gynecol.

[22] Yu D, Zhang K, Han M, Pan W, Chen Y, Wang Y (2019). Noninvasive prenatal testing for fetal subchromosomal copy number variations and chromosomal aneuploidy by low-pass whole-genome sequencing. Mol Genet Genomic Med.

[23] Pös O, Budis J, Kubiritova Z, Kucharik M, Duris F, Radvanszky J (2019). Identification of structural variation from NGS-based non-invasive prenatal testing. Int J Mol Sci.

[24] Hyblova M, Harsanyova M, Nikulenkov-Grochova D, Kadlecova J, Kucharik M, Budis J (2020). Validation of copy number variants detection from pregnant plasma using low-pass whole-genome sequencing in noninvasive prenatal testing-like settings. Diagnostics (Basel)..

[25] Ye X, Lin S, Song X, Tan M, Li J, Wang J (2021). Identification of copy number variants by NGS-based NIPT at low sequencing depth. Eur J Obstet Gynecol Reprod Biol.

[26] Srebniak MI, Vogel I, Van Opstal D (2018). Is carriership of a balanced translocation or inversion an indication for non-invasive prenatal testing?. Expert Rev Mol Diagn.

[27] Raman L, Dheedene A, De Smet M, Van Dorpe J, Menten B (2019). WisecondorX: improved copy number detection for routine shallow whole-genome sequencing. Nucleic Acids Res.

[28] Wilkinson L (2011). ggplot2: Elegant graphics for data analysis by WICKHAM, H. Biometrics.

[29] Yin T, Cook D, Lawrence M (2012). ggbio: an R package for extending the grammar of graphics for genomic data. Genome Biol.

[30] García-Pascual CM, Navarro-Sánchez L, Navarro R, Martínez L, Jiménez J, Rodrigo L (2020). Optimized NGS approach for detection of aneuploidies and mosaicism in PGT-A and imbalances in PGT-SR. Genes (Basel)..

[31] Wang JC, Sahoo T, Schonberg S, Kopita KA, Ross L, Patek K (2015). Discordant noninvasive prenatal testing and cytogenetic results: a study of 109 consecutive cases. Genet Med.

[32] Miller DT, Adam MP, Aradhya S, Biesecker LG, Brothman AR, Carter NP (2010). Consensus statement: chromosomal microarray is a first-tier clinical diagnostic test for individuals with developmental disabilities or congenital anomalies. Am J Hum Genet.

[33] Levy B, Wapner R (2018). Prenatal diagnosis by chromosomal microarray analysis. Fertil Steril.

[34] Osborne CM, Hardisty E, Devers P, Kaiser-Rogers K, Hayden MA, Goodnight W (2013). Discordant noninvasive prenatal testing results in a patient subsequently diagnosed with metastatic disease. Prenat Diagn.

[35] Bianchi DW, Parsa S, Bhatt S, Halks-Miller M, Kurtzman K, Sehnert AJ (2015). Fetal sex chromosome testing by maternal plasma DNA sequencing: clinical laboratory experience and biology. Obstet Gynecol.

[36] Ji X, Chen F, Zhou Y, Li J, Yuan Y, Mo Y (2018). Copy number variation profile in noninvasive prenatal testing (NIPT) can identify co-existing maternal malignancies: case reports and a literature review. Taiwan J Obstet Gynecol.

[37] Wang T, Duan C, Shen C, Xiang J, He Q, Ding J (2016). Detection of complex deletions in chromosomes 13 and 21 in a fetus by noninvasive prenatal testing. Mol Cytogenet.

[38] Mei J, Wang H, Zhan L (2017). 10p15.3p13 duplication inherited from paternal balance translocation (46, XY, t(5;10)(q35.1;p13)) identified on non-invasive prenatal testing. J Obstet Gynaecol Res.

[39] Chen JK, Liu P, Hu LQ, Xie Q, Huang QF, Liu HL (2018). A foetus with 18p11.32-q21.2 duplication and Xp22.33-p11.1 deletion derived from a maternal reciprocal translocation t(X;18)(q13;q21.3). Mol Cytogenet.

[40] Zheng Y, Chen B, Wan S, Xu H, Dang Y, Song T (2019). Detection of 21q11.2–q22.11 deletions in a fetus by NIPT. J Clin Lab Anal.

[41] Liu T, Xie H, Zhang J, Wang X, Sha J, Zhai J (2020). Fetus of 8q22.2q24.3 duplication and 13q33.2q34 deletion derived from a maternal balanced translocation. J Obstet Gynaecol Res.

[42] Yin L, Tang Y, Lu Q, Pan A, Shi M (2020). Application value of NIPT for uncommon fetal chromosomal abnormalities. Mol Cytogenet.

[43] American College of Medical Genetics and Genomics (2010). Standards and Guidelines for Clinical Genetics Laboratories Clinical. E: Cytogenetics.

[44] Zhao G, Dai P, Gao S, Zhao X, Wang C, Liu L (2019). A case of prenatal diagnosis of 18p deletion syndrome following noninvasive prenatal testing. Mol Cytogenet.

[45] Qian YQ, Wang XQ, Chen M, Luo YQ, Yan K, Yang YM (2019). Detection of fetal subchromosomal aberration with cell-free DNA screening led to diagnosis of parental translocation: Review of 11344 consecutive cases in a university hospital. Eur J Med Genet.

[46] Silva M, de Leeuw N, Mann K, Schuring-Blom H, Morgan S, Giardino D (2019). European guidelines for constitutional cytogenomic analysis. Eur J Hum Genet.

[47] Arıkan DC, Coşkun A, Arıkan I, Kıran G, Ceylaner G (2010). Prenatally diagnosed partial trisomy 3q case with an omphalocele and less severe phenotype. J Turk Ger Gynecol Assoc.

[48] Imataka G, Watabe Y, Kajitani S, Watanabe S, Ichikawa J, Drago F (2017). Rare de novo inversion-duplication case with pure 3qter duplication syndrome including an overlap of the dup(3q) critical region: a case report. Rare Exp Ther Med.

[49] Rossi M, Di Micco P, Perone L, De Brasi D, Guzzetta V, Andreucci MV (2002). Unbalanced translocation (3;5)(q26.1;p14): a clinical report. Am J Med Genet.

[50] Pasińska M, Adamczak R, Repczyńska A, Łazarczyk E, Iskra B, Runge AK (2019). Prenatal identification of partial 3q duplication syndrome. BMC Med Genomics.

[51] Aqua MS, Rizzu P, Lindsay EA, Shaffer LG, Zackai EH, Overhauser J (1995). Duplication 3q syndrome: molecular delineation of the critical region. Am J Med Genet.

[52] Krantz ID, Tonkin E, Smith M, Devoto M, Bottani A, Simpson C (2001). Exclusion of linkage to the CDL1 gene region on chromosome 3q26.3 in some familial cases of Cornelia de Lange syndrome. Am J Med Genet.

[53] Lisi EC, Hamosh A, Doheny KF, Squibb E, Jackson B, Galczynski R (2008). 3q29 interstitial microduplication: a new syndrome in a three-generation family. Am J Med Genet A.

[54] Abreu-González M, García-Delgado C, Cervantes A, Aparicio-Onofre A, Guevara-Yáñez R, Sánchez-Urbina R (2013). Clinical, cytogenetic, and biochemical analyses of a family with a t(3;13)(q26.2;p11.2): further delineation of 3q duplication syndrome. Case Rep Genet..

[55] Gu H, Jiang JH, Li JY, Zhang YN, Dong XS, Huang YY (2013). A familial Cri-du-Chat/5p deletion syndrome resulted from rare maternal complex chromosomal rearrangements (CCRs) and/or possible chromosome 5p chromothripsis. PLoS ONE.

[56] Flowers NJ, Burgess T, Giouzeppos O, Shi G, Love CJ, Hunt CE (2020). Genome-wide noninvasive prenatal screening for carriers of balanced reciprocal translocations. Genet Med.

